# Functional analysis of molecular and pharmacological modulators of mitochondrial fatty acid oxidation

**DOI:** 10.1038/s41598-020-58334-7

**Published:** 2020-01-29

**Authors:** Yibao Ma, Wei Wang, Teja Devarakonda, Huiping Zhou, Xiang-Yang Wang, Fadi N. Salloum, Sarah Spiegel, Xianjun Fang

**Affiliations:** 1 Departments of Biochemistry & Molecular Biology, Virginia Commonwealth University School of Medicine, Richmond, Virginia 23298 USA; 2 Internal Medicine/Cardiology Pauley Heart Center, Virginia Commonwealth University School of Medicine, Richmond, Virginia 23298 USA; 3 Microbiology & Immunology, Virginia Commonwealth University School of Medicine, Richmond, Virginia 23298 USA; 4 Human & Molecular Genetics, Virginia Commonwealth University School of Medicine, Richmond, Virginia 23298 USA

**Keywords:** Biochemistry, Biological techniques, Cancer

## Abstract

Fatty acid oxidation (FAO) is a key bioenergetic pathway often dysregulated in diseases. The current knowledge on FAO regulators in mammalian cells is limited and sometimes controversial. Previous FAO analyses involve nonphysiological culture conditions or lack adequate quantification. We herein described a convenient and quantitative assay to monitor dynamic FAO activities of mammalian cells in physiologically relevant settings. The method enabled us to assess various molecular and pharmacological modulators of the FAO pathway in established cell lines, primary cells and mice. Surprisingly, many previously proposed FAO inhibitors such as ranolazine and trimetazidine lacked FAO-interfering activity. In comparison, etomoxir at low micromolar concentrations was sufficient to saturate its target proteins and to block cellular FAO function. Oxfenicine, on the other hand, acted as a partial inhibitor of FAO. As another class of FAO inhibitors that transcriptionally repress FAO genes, antagonists of peroxisome proliferator-activated receptors (PPARs), particularly that of PPARα, significantly decreased cellular FAO activity. Our assay also had sufficient sensitivity to monitor upregulation of FAO in response to environmental glucose depletion and other energy-demanding cues. Altogether this study provided a reliable FAO assay and a clear picture of biological properties of potential FAO modulators in the mammalian system.

## Introduction

Fatty acid oxidation (FAO) is a key catabolic pathway for energy production in mammals^1^. Long-chain fatty acids are first activated in the cytosol to fatty acyl-CoAs. Fatty acyl-CoAs are transported by the carnitine shuttle system into the mitochondrion where they undergo multi-step reactions to generate acetyl-CoA which can be further oxidized through the Krebs cycle. The reduced electron carriers FADH_2_ and NADH+H^+^ from FAO and the Krebs cycle deliver electrons to the electron transport chain (ETC) to produce ATP through oxidative phosphorylation. In humans, FAO is a predominant bioenergetic source, accounting for more than half of total ATP production^1^. In addition, FAO-generated acetyl-CoA is involved in production of cytosolic NADPH^2–5^, providing the reducing power to support biosynthesis and to counteract oxidative stress.

FAO is dysregulated in numerous diseases^1,6^. In particular, as a result of insufficient insulin sensitivity and limited glucose oxidation, diabetic patients show hyperactive FAO and ketogenesis, a process that diverts FAO-derived acetyl-CoA to ketone bodies in the liver^7^. Recently, emerging evidence suggests that FAO is abnormally upregulated in cancer in connection with oncogenic proteins such as c-Myc^8^, STAT-3^9^, and c-Src^10^. FAO is required for the maintenance of the malignant phenotype of cancer^4^. However, the molecular mechanisms regulating FAO activities under various pathophysiological conditions remain obscure^4,11^.

Although many chemical compounds are considered to be inhibitors or activators of FAO enzymes^4^, the current knowledge on their specificities, potencies and metabolic impacts is very limited and sometimes controversial. Many previous conclusions have been based on the effects of these compounds on enzymatic activities, abundances of metabolic intermediates, or surrogate parameters of cellular FAO activities^10,12–17^. This has led to inconsistent information regarding biological activities of a variety of potential FAO-modulating agents. For instance, etomoxir, the best-known inhibitor of carnitine palmitoyltransferase 1 (CPT1), has been used in different studies from micromolar to millimolar concentrations^2,17–20^. Apparently not all cellular effects of this wide range of concentrations are attributable to specific inhibition of FAO. Indeed, recent studies have demonstrated off-target effects of etomoxir independent of CPT1-supported FAO, such as inhibition of Complex I of ETC^18^ and promotion of ROS production^21^. Other such examples are the so-called metabolic modifiers including ranolazine (Ranexa) and trimetazidine (TMZ) currently used in clinic for treatment of angina pectoris^22^. They were assumed to be partial FAO inhibitors that targeted 3-ketoacyl-CoA thiolase (3-KAT), a component of the trifunctional protein (TFP) (hydroxyacyl-CoA dehydrogenase/enoyl-CoA hydratase/3-KAT)^23,24^. The TFP complex catalyzes the last three steps of β-oxidation within the mitochondrion^1^. However, the anti-FAO properties of ranolazine and TMZ have not been thorougly characterized. Recent studies revealed that these drugs more likely interfere with other cellular functions such as mitochondrial calcium and sodium channels to exert their clinical benefits^25–30^.

A common method for direct measurement of FAO quantifies the conversion of [9,10-^3^H(N)]-palmitic acid to ^3^H_2_O^20,31,32^. Conventionally, the method requires physical separation of ^3^H_2_O from unoxidized ^3^H-palmitic acid. For the convenience of lipid extraction, precipitation and column separation of ^3^H-palmitic acid from ^3^H_2_O, cells are usually labeled with ^3^H-palmitic acid in biological buffers such as the Krebs buffer to avoid the heavy lipid and protein contents present in regular serum-containing medium. The artificial labeling solutions contain limited concentrations of palmitic acid as the sole FAO substrate. In this condition, cells are forced to rely more on FAO in the absence of amino acids and other nutrients. In addition, cellular FAO activities are also affected by proliferative status of cells and FAO regulators present in complete medium.

In the present study, we examined the possibility that ^3^H_2_O could be “separated” from un-oxidized ^3^H-palmitic acid by diffusion to quantify ^3^H_2_O production in physiologically relevant culture and *in vivo* settings. Some recent studies have applied the diffusion method for FAO assay^33,34^. However, several technical issues remain to be resolved. First, water-soaked 3 M paper instead of a fixed volume of aqueous solution was used to absorb ^3^H_2_O from culture supernatants. Given the uncontrolled amounts of water in the 3 M paper and the unknown ratios to the volumes of culture supernatants used for the assay, it was not possible to determine the exact conversion rate from ^3^H-palmitic acid to ^3^H_2_O. Secondly, the assay ignored the weak volatility of fatty acids and their aliphatic metabolites^35^ that could also attach to the 3 M paper, causing overestimation of the results. These limitations together make the described vapor method a hardly quantitative assay for functional analysis of FAO. We therefore improved the assay by setting up the diffusion between two liquid phases of controlled volumes in an airtight tube. The weak volatility of ^3^H-palmitic acid was determined with a cell-free control group in order to be excluded from the measurements. We took advantage of the improved method to systematically assess activities of multiple molecular and pharmacological regulators of the FAO pathway.

## Results

### A convenient and quantitative FAO assay

To improve the current methods for FAO measurement, we examined the possibility that ^3^H_2_O released from FAO could be separated from ^3^H-palmitic acid and its metabolic intermediates through water diffusion in an airtight tube. In this scenario, the cells could be labeled in complete serum-containing medium that provides growth factors, amino acids, various fatty acids and other nutrients so we can assess cellular FAO activities in physiologically more relevant conditions and compare *“in situ*” effects of FAO-modulating agents. We and others have used a similar method to “separate” ^3^H_2_O from non-diffusible glucose^36,37^. We hypothesized that the limited volatility of palmitic acid and its metabolites can be excluded from results by introducing a cell-free blank control.

**Figure 1:**
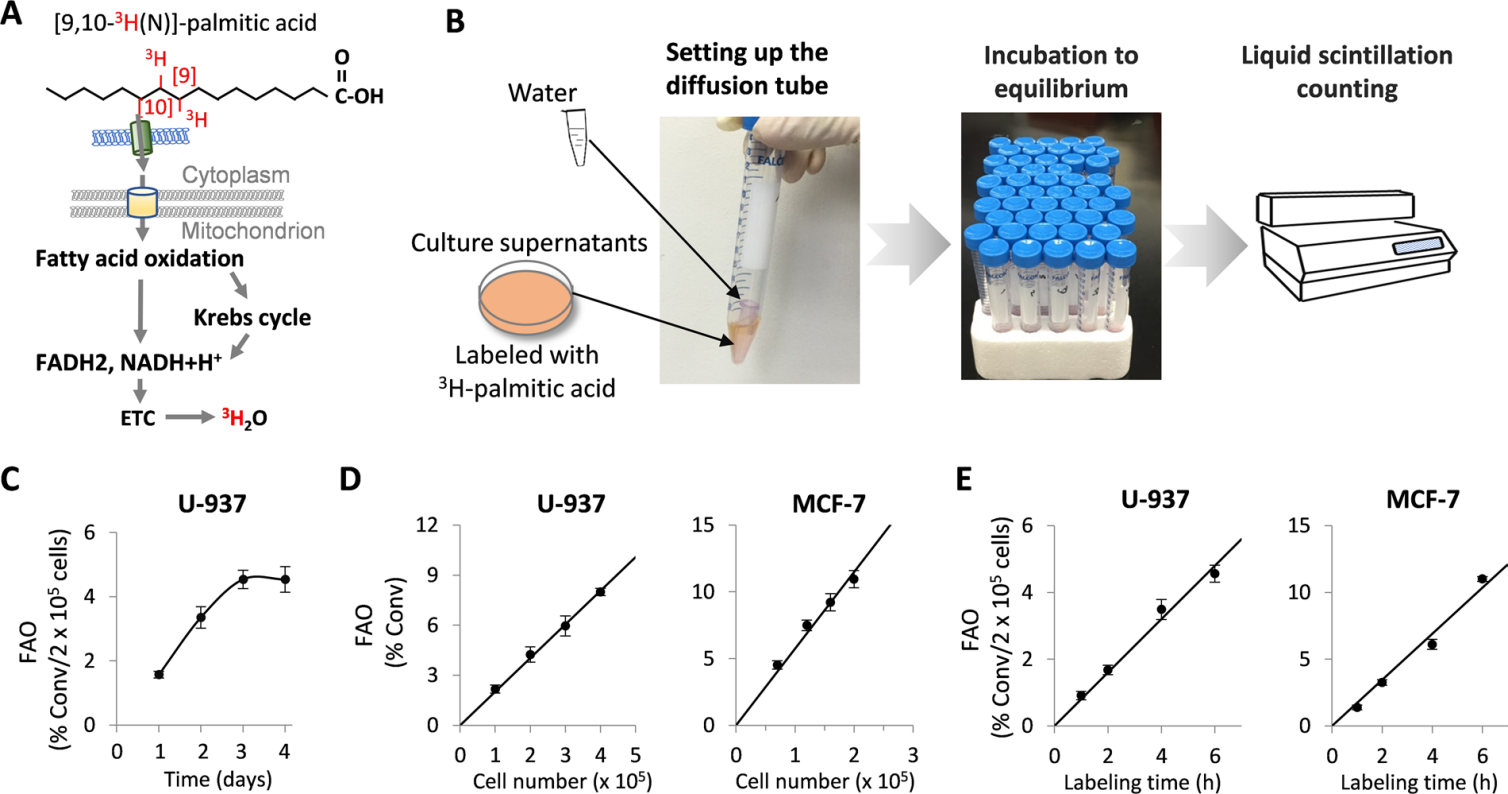
Development of an FAO quantification assay. (**A**) The process of converting [9,10-^3^H]-palmitic acid to ^3^H_2_O via the mitochondrial FAO is illustrated. (**B**) After labeling in complete medium with [9,10-^3^H]-palmitic acid, culture supernatants were collected into 15-ml Falcon centrifuge tubes. With the aid of forceps, an uncapped microcentrifuge tube containing 0.25 ml of sterile distilled water was inserted into the Falcon tube. The 15-ml tube was then tightly closed and left at room temperature to start diffusion. After reaching equilibrium, the radioactivities present in the two phases were read by liquid scintillation counting and FAO rates calculated as described in Materials and Methods. (**C**) To determine the diffusion time required for reaching equilibrium, culture supernatants from U-937 cells labeled with ^3^H-palmitic acid were incubated for 1, 2, 3, or 4 days. The FAO rates presented as % conversion (conv)/2 × 10^5^ cells/5 hours were plotted as function of incubation time (days). (**D**) To assess the range of cell numbers suitable for the assay, FAO rates were determined after 5 hours of ^3^H-palmitic acid labeling of increasing numbers of U-937 cells (1–4 × 10^5^, *left*) or MCF-7 cells (0.5–2 × 10^5^, *right*). (***E***) Fixed numbers (2 × 10^5^) of U-937 and MCF-7 cells were subjected to different labeling time (1, 2, 4, 6 hours). FAO rates were plotted as function of labeling intervals.

Initially, we selected the U-937 monocytic lymphoma and MCF-7 breast carcinoma cell lines representing FAO-active suspension and adherent cell types, respectively^38,39^. We first determined the optimal diffusion time that allowed the detection of ^3^H_2_O in culture medium from U-937 cells. Following labeling with ^3^H-palmitic acid in complete culture medium, the supernatants were collected to set the diffusion device as shown in Fig. 1A,B. After 1-4 days of incubation at room temperature and conversion rates were calculated as detailed in Materials and Methods. For each time point, a control group to trace weakly diffused ^3^H-palmitic acid in the absence of cells was analyzed in parallel. The average background rate from diffused ^3^H-palmitic acid, usually ranging from 0.5% to 2.6%, was subtracted from each experimental sample. Since medium pH might affect ionization states of fatty acids and therefore their volatilities, we examined if 0.2N NaOH instead of HCl added to terminate labeling might eliminate the background from fatty acid diffusion. As shown in Supplementary Fig. S1A, the background reading in the base condition was partially reduced from 2.17% to 1.22% but remained significant.

As shown in Fig. 1C, the measured FAO rates increased with incubation time, reaching a plateau by 3 days. Beyond that, the readings became stable or started to drop slightly, reflecting the equilibrium of water diffusion by 3 days. The data indicates that the diffusion method is sufficient to distinguish the radioactivity of ^3^H_2_O from that of weakly volatile palmitic acid and aliphatic metabolites.

We next examined whether the measurement of ^3^H_2_O indeed reflected cellular FAO activity by comparing the rates obtained from different numbers of cells. Thus, 1–4 × 10^5^ of U-937 or 0.5–2 × 10^5^ of MCF-7 cells in 12-well plates were labeled with ^3^H-palmitic acid for 5 hours before supernatants were harvested for measuring FAO. As shown in Fig. 1D, the obtained FAO rates directly correlated with the numbers of U-937 or MCF-7 cells used for the experiment. To determine the optimal labeling time, fixed numbers of U-937 and MCF-7 cells were labeled for 1, 2, 4 or 6 hours. The measured FAO rates increased linearly with the labeling time as shown in Fig. 1E. These results indicate that this improved assay is suitable for a wide range of cell numbers and flexible labeling times.

Most previous FAO assays analyzed medium only, excluding the cells from the measurement^32,34,40,41^. This might cause overestimation of FAO if intracellular ^3^H-palmitic acid contents are significant or vary dramatically among various cell types. We therefore compared the results from analysis of only culture supernatants with that covering both medium and cells. The results shown in Supplementary Fig. S1B indicated that the measured FAO rate was indeed slightly higher if only culture supernatants were analyzed. However, this caused only minor and systematic differences that did not influence the comparison of groups or experimental conclusions. For example, treatment of cells with etomoxir, a prototype inhibitor of CPT1, significantly decreased FAO rates to a similar extent regardless of supernatants only or combination of cells and supernatants to be assayed (64% versus 67% in U-937, and 73% versus 75% in MCF-7). Therefore, for the remainder of the study, we analyzed culture supernatants only although the method was fully amenable to including cells in the measurement.

We further compared FAO activities in cells labeled in complete culture medium or in Krebs buffer. As shown in Supplementary Fig. S2A, the cells labeled in Krebs buffer tended to display significantly higher basal FAO activities in both MCF-7 and T47D cells, consistent with the presumption that the cells turn to more active FAO in the artificial buffer. Etomoxir seemed to work more efficaciously to block FAO in the buffer than in the complete medium. This may be related to the better accessibility of the compound to cells in the simple buffer solution than in the more complicated medium. To further validate our assay, we compared our diffusion assay with the conventional method to physically separate ^3^H_2_O from ^3^H-palmitic acid^40^. MCF-7 and T47D cells were labeled in Krebs buffer and analyzed with these two approaches in parallel. As shown in Supplementary Fig. S2B, the two methods yielded essentially similar results except that our method detected slightly higher FAO rates in both cell lines.

### Validation of the FAO assay with molecular approaches

**Figure 2:**
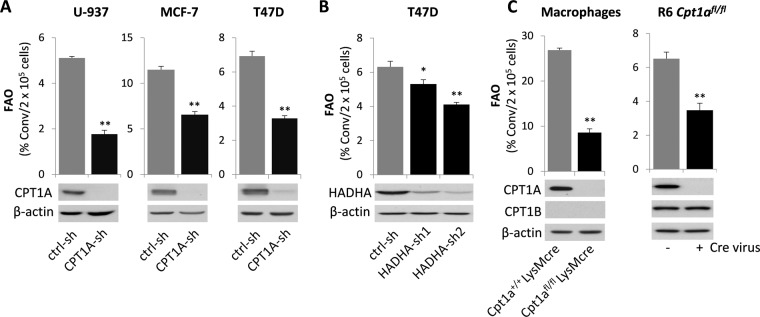
Validation of the FAO assay with molecular and genetic approaches. (**A**) CPT1A of the FAO pathway was knocked down in U-937, MCF-7, and T47D cells with lentivirus-mediated CPT1A-sh RNA. The effects of CPT1A silencing on FAO rates in these knockdown and control cells were determined. The labeling time was 5 hours hereinafter, unless otherwise specified. (**B**) The expression of HADHA of TFP was silenced in T47D cells with two independent HADHA-sh RNAs to determine the consequence on FAO rates. (**C**) Bone marrow cells were collected from *Cpt1a*^*+/+*^*LysMcre* and *Cpt1a*^*fl/fl*^ *LysMcre* mice. After the cells were differentiated into macrophages in culture, FAO rates were measured and compared between the *Cpt1a* WT and KO macrophages (*left*). The loxped *Cpt1a* was homozygously deleted from the R6 *Cpt1a*^*fl/fl*^ MEF line with *Cre* virus before FAO rates were measured in these *Cpt1a* WT and KO fibroblasts (*right*). Full-length blots are presented in Supplementary Fig. S4.

To confirm the reliability of our FAO quantification method, we tested its capability to detect FAO changes as a result of molecular downregulation or genetic disruption of key FAO enzymes. CPT1 is the rate-limiting enzyme responsible for shuttling fatty acyl-CoA into the mitochondrion for FAO^1^. We knocked down CPT1A in U-937, MCF-7 and T47D cells with lentivirus-transduced CPT1A-shRNA. The FAO activity was significantly reduced in CPT1A-silenced cells compared to the control counterparts (Fig. 2A), consistent with an essential role for CPT1A in fueling FAO in these cells. The hydroxyacyl-CoA dehydrogenase trifunctional protein (TFP) complex is comprised of four α subunit (HADHA) and four β subunits (HADHB)^1^. Knockdown of HADHA in T47D cells also significantly decreased FAO activity (Fig. 2B). The high residual FAO levels likely resulted from limited knockdown efficiency, the presence of CPT1B, or the contribution of peroxisomal oxidation.

As a genetic cell model deficient in *Cpt1a*, we isolated bone marrow cells from *Cpt1a*^*fl/fl*^*LysMcre* mice^42^ and their wild type littermates *Cpt1a*^*+/+*^*LysMcre*. After differentiation into macrophages in culture, FAO was measured. The metabolism of ^3^H-palmitic acid to ^3^H_2_O was dramatically decreased in the *Cpt1a* deficient macrophages compared to that in control macrophages (Fig. 2C). In addition, the mouse embryonic fibroblast (MEF) line R6 *Cpt1a*^*fl/fl*^ was infected with the lentivirus pCDH-Cre to allow homozygous deletion of *Cpt1a*. The loss of *Cpt1a* in MEFs also significantly decreased FAO activity (Fig. 2C). Only a partial decrease instead of more complete abrogation was likely due to the presence of CPT1B protein in the immortalized MEFs (Fig. 2C).

### Assessment of putative FAO inhibitors

A large number of compounds have been considered to be inhibitors of FAO enzymes or FAO activity. Among them, etomoxir^43^ and oxfencine^44,45^ are inhibitors of CPT1. The anti-angina drugs perhexiline, ranolazine, and TMZ are considered to be partial inhibitors of FAO. Perhexiline is a potential CPT1/CPT2 dual inhibitor^46,47^ whereas ranolazine and TMZ are expected to target 3-KAT of the TFP complex^23,24^. Although they may indeed inhibit specific FAO enzymes, most of these compounds except etomoxir haven’t been appropriately evaluated for their anti-FAO functions on the cellular level.

**Figure 3:**
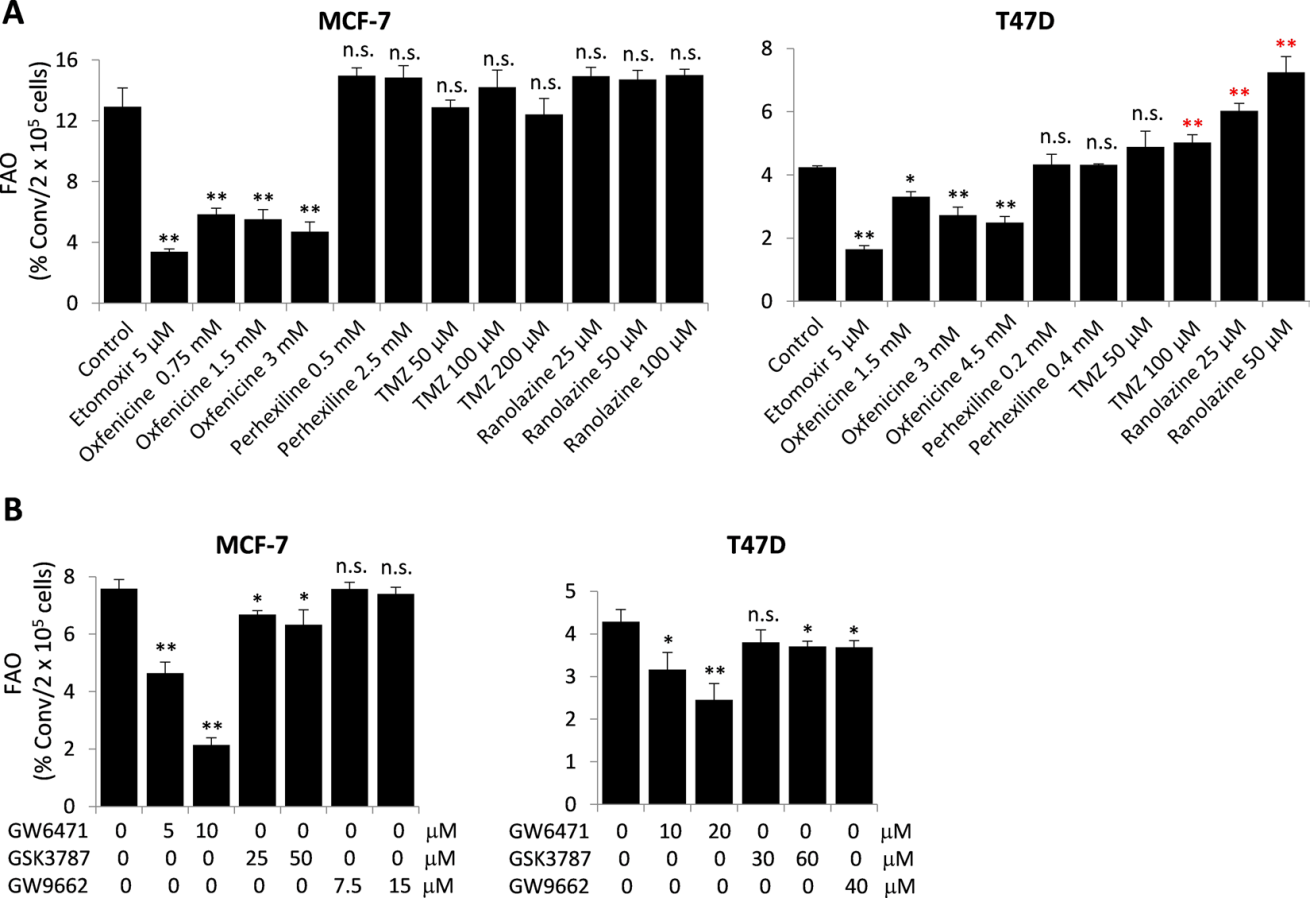
Assessment of anti-FAO activities of putative FAO inhibitors. MCF-7 and T47D cells were treated with the CPT inhibitors (etomoxir, oxfenicine and perhexiline) or potential 3-KAT inhibitors (TMZ and ranolazine) at indicated concentrations for 24 hours (**A**). In (**B**), the cells were treated with the antagonists of PPARα (GW6471), PPARβ/δ (GSK3787) or PPARγ (GW9662) at indicated concentrations for 30 hours before FAO measurement.

We therefore used our new FAO quantification assay to assess their potential anti-FAO activities with etomoxir as a positive control. Surprisingly, we found that besides etomoxir, only oxfenicine showed significant inhibition of FAO in MCF-7 and T47D cells (Fig. 3A). The effective dose of oxfenicine was much higher than that of etomoxir. At 3 mM, oxfenicine decreased FAO rates by 36% and 64% in T47D and MCF-7 cells, respectively. None of the other compounds (perhexiline, ranolazine or TMZ) significantly inhibited FAO in either MCF-7 or T47D cells. On the contrary, these compounds at high concentrations modestly increased FAO specifically in T47D cells.

Since the lack of the FAO-inhibitory effect of ranolazine and TMZ was unexpected, we turned to the ^14^CO_2_ capture method to compare with our assay and to confirm the findings on these drugs. As shown in Supplementary Fig. S3, the % ^14^CO_2_ conversion rates measured from the ^14^CO_2_ capture method were much lower than those obtained from our ^3^H_2_O diffusion method. The observation was in agreement with the contention that only a small proportion of the acetyl residues produced by FAO were converted immediately to CO_2_ with the remainder being incorporated into non-volatile metabolic intermediates^48^. However, the results from these two independent methods were consistent with respect to the conclusions on the effects of etomoxir, ranolazine, and TMZ on FAO.

The peroxisome proliferator-activated receptors (PPARs) of the ligand-activated nuclear receptor superfamily are the most prominent transcriptional regulators of FAO enzymes^49,50^. They act essentially as environmental fat sensors and activators of FAO^41,51,52^. Antagonists of PPARα, PPARβ/δ and PPARγ have been reported to inhibit FAO in different experimental settings^41,52,53^. To gain better understanding of PPAR regulation of FAO, we assessed the effects of various PPAR antagonists. In MCF-7 and T47D cells, FAO rates were most dramatically suppressed by the PPARα antagonist GW6471 (Fig. 3B). Relatively weaker inhibition of FAO was seen with the PPARβ/δ antagonist GSK3787. The PPARγ antagonist GW9662 was toxic towards MCF-7 cells. At non-toxic concentrations, GW9662 did not affect FAO in MCF-7 cells. At a relatively high dose (40 μM), it partially inhibited FAO in T47D cells (Fig. 3B).

### Analysis of biologically relevant concentrations of etomoxir

A broad range of concentrations of etomoxir have been reported to inhibit FAO activities in different types of cells^2,17–20^. This has led to the question of whether diverse biological functions of etomoxir are indeed resulted from FAO inhibition rather than tampering with other cellular targets. The concern is further highlighted by recent studies showing that relatively high concentrations of etomoxir inhibited Complex I of ETC^18^, activated PPARα^54,55^, and promoted ROS production^21^ independently of the CPT1-FAO axis.

**Figure 4:**
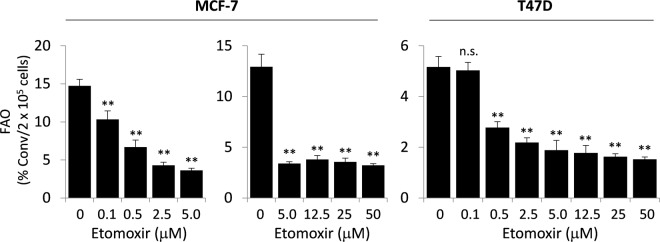
Biologically relevant concentrations of etomoxir for FAO inhibition. FAO rates were quantified in MCF-7 and T47D cells treated for 24 hours with etomoxir from 0.1 to 50 μM concentrations. The results are presented as in Fig. 1C.

To clarify the potency of etomoxir as a specific FAO inhibitor, we treated MCF-7 and T47D cells with 0.1 to 50 μM of etomoxir. In both cell lines, etomoxir at 0.5 μM significantly decreased FAO rates as shown in Fig. 4. The effect of etomoxir reached a maximum at approximately 5 μM in MCF-7 (76% inhibition) and 5-12.5 μM in T47D (66% inhibition). These results clearly showed that low micromolar concentrations of etomoxir were sufficient to achieve maximal inhibition of FAO in MCF-7 and T47D cells. Similar results were observed in U-937 (Supplementary Fig. S1B) and MDA-MB-468 cells (data not shown).

### Potency of etomoxir binding to cellular CPT1A protein

**Figure 5:**
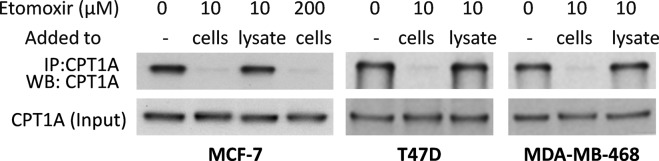
Potency of etomoxir binding to cellular CPT1A protein. MCF-7, T47D, and MDA-MB-468 cells treated for 20–24 hours with etomoxir at indicated concentrations were analyzed with immunoprecipitation of native CPT1A protein and immunoblotting of denatured lysates for CPT1A (input) with the same CPT1A antibody. Etomoxir added to intact cells, but not to cellular lysates, prevented CPT1A protein from being immunoprecipitated by the CPA1A antibody. Full-length blots are presented in Supplementary Fig. S4.

Etomoxir is a prodrug that is converted intracellularly to the active etomoxir-CoA ester^56^. The latter irreversibly binds to CPT1 to inhibit FAO. Given the fact that a low micromolar concentration of exogenously added etomoxir exerted maximal FAO-inhibitory activity, we speculated that this dose would lead to saturated binding of etomoxir-CoA to cellular CPT1A protein. To gain the molecular evidence for this, we examined whether etomoxir-CoA binding to CPT1A could prevent immunoprecipitation of the protein by certain CPT1A antibodies. We examined several anti-CPT1A antibodies to search for one that could compete with etomoxir-CoA for binding to the C-terminus of CPT1A protein. Fortunately, one monoclonal antibody generated against a C-terminal fragment of human CPT1A (amino acids 489–773) (Abcam ab128568) was found to immunoprecipitate CPT1A protein efficiently from MCF-7, T47D and MDA-MB-468 cells. Treatment of these cells with 10 μM etomoxir nearly completely prevented the protein from being precipitated by the antibody (Fig. 5), indicating that CPT1A protein in etomoxir-treated cells was mostly occupied and sequestered by etomoxir-CoA. In further support of the specific interaction between etomoxir-CoA and CPT1A, etomoxir itself failed to prevent the immunoprecipitation when added directly to cell lysates instead of treatment of intact cells. In addition, the effects were similar in MCF-7 cells treated with either 10 or 200 μM etomoxir (Fig. 5), further supporting the conclusion that low micromolar etomoxir provides a sufficient level of etomoxir-CoA to dominate CPT1A protein.

### Regulation of FAO by carnitine, catabolic stimulants and nutrient status

**Figure 6:**
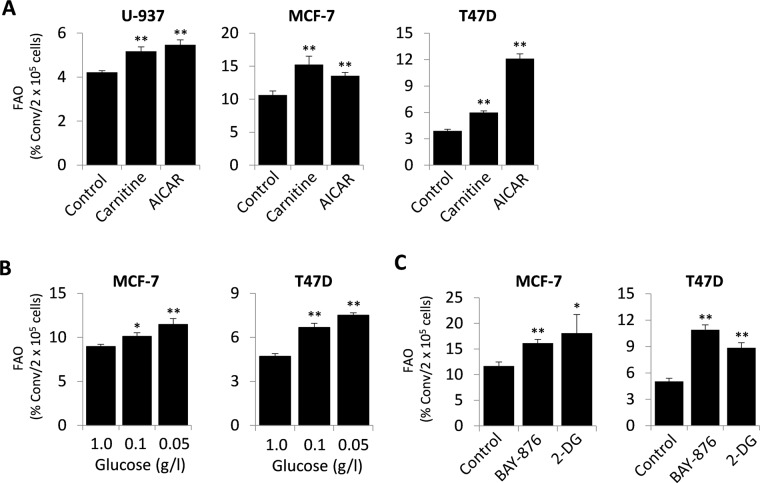
Induction of FAO by FAO activators. (**A**) FAO rates were measured in U-937, MCF-7, and T47D cells treated for 24 hours with the CPT1 substrate carnitine (0.5 mM) or with the AMPK activator AICAR (1 mM). FAO activities were also determined in MCF-7 and T47D cells grown under decreasing concentrations of glucose (1.0, 0.1 and 0.05 g/l) (**B**), or treated with the GLUT1 inhibitor BAY-876 (75 nM) or with the hexokinase inhibitor 2-DG (2 mM) (**C**) for 24 hours.

We next tested the effects of potential FAO activators. Carnitine, as an essential substrate of CPT1, has been used as a driver of cellular FAO activities when exogenously supplied^13^. Indeed, the FAO-stimulating action of carnitine (0.5 mM) was readily detectable (Fig. 6A), implying that carnitine levels should be maintained consistent in a given experiment to avoid any bias in the assessment of FAO activities. The compound 5-aminoimidazole-4-carboxamide ribonucleoside (AICAR) is a cell-permeable AMP analog that stimulates FAO via activation AMP-activated kinase (AMPK) and AMPK-mediated phosphorylation and inactivation of acetyl-CoA carboxylase (ACC)^57,58^. ACC catalyzes the synthesis of malonyl-CoA, an allosteric inhibitor of CPT1^58^. Indeed, AICAR (1 mM) significantly increased FAO activity in these cell lines (Fig. 6A).

Cellular FAO activity is physiologically regulated by the nutrient status, most prominently by the availability of glucose which is the preferred source for energy production in many cell types^59^. When glucose catabolism is limited, FAO is supposed to be adaptively activated. We tested this potential regulation of FAO by minimizing medium glucose concentrations from 1 g/l to 0.05 g/l. This led to a gradual elevation of FAO as shown in Fig. 6B. Similarly, when glucose metabolism was inhibited by BAY-876, a new inhibitor of glucose transporter I^60,61^, or by 2-deoxyglucose (2-DG), an inhibitor of hexokinases^62^, MCF-7 and T47D cells also responded with increases in FAO activities (Fig. 6C).

### Effects of FAO inhibitors in primary cells and in mice

**Figure 7:**
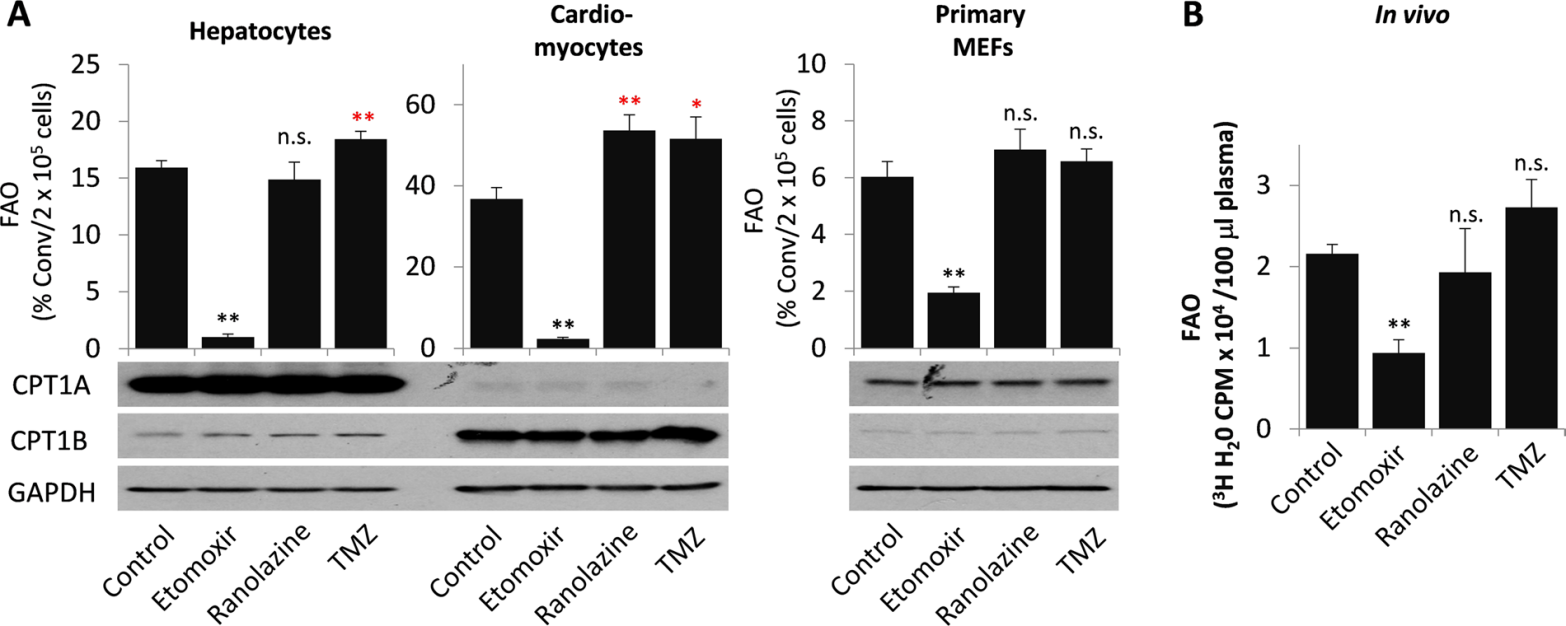
Effects of etomoxir, ranolazine and TMZ in primary cells and in mice (**A**) FAO rates in primary rat hepatocytes, mouse cardiomyocytes, and MEFs treated for 20–24 hours with or without etomoxir (5 μM), ranolazine (25 μM), or TMZ (50 μM) were measured as detailed in Materials and Methods. The predominant expression of tissue-specific CPT1A (liver form) in hepatocytes and CPT1B (muscle form) in cardiomyocytes was confirmed by immunoblotting. Full-length blots are presented in Supplementary Fig. S4. The FAO rates in the metabolically active hepatocytes and cardiomyocytes are presented as % conv/2 × 10^5^ cells/2 hours while those in MEFs were presented as % conv/2 × 10^5^ cells/5 hours. (**B**) Four groups of adult female mice (n = 3 each group) were treated with etomoxir, ranolazine or TMZ for 3 days before administration of ^3^H-palmitic acid as detailed in Materials and Methods. The production of ^3^H_2_O from *in vivo* oxidation of ^3^H-palmitic acid over 1-hour period was assessed by the diffusion analysis of plasma samples of mice. The results were presented as ^3^H_2_O-based radioactivity (CPM)/100 µl plasma.

Physiologically, FAO is extremely active in the high energy-demanding heart and the liver, the central organ of lipid metabolism^1^. Hepatocytes and cardiomyocytes thus represent physiologically appropriate cell models to study FAO activity and regulation. We next used primary rat hepatocytes and mouse cardiomyocytes to assess the feasibility of the assay to measure metabolically active FAO and the responses of these cells to potential FAO inhibitors. To maintain the differentiated phenotypes, these primary cells were maintained in culture for less than 24 hours with the last 2 hours of labeling with ^3^H-palmitic acid. The short labeling was chosen for the metabolic active hepatocytes and cardiomyocytes to ensure linear reaction over the labeling duration. As shown in Fig. 7A, high basal FAO rates were detected in primary hepatocytes and cardiomyocytes. Consistent with the earlier observations in established cell lines, the FAO activities in these primary cells were strongly inhibited by etomoxir but not by ranolazine or TMZ. Consistent with the effects of these compounds in T47D cells (Fig. 3A), TMZ slightly increased FAO in both mouse cardiomyocytes and rat hepatocytes while ranolazine exhibited such an effect only in rat hepatocytes (Fig. 7A). Furthermore, we measured FAO activity in freshly isolated MEFs as another example of primary cells. The basal FAO activity was relatively low in primary MEFs. The MEF FAO activity was sensitive to etomoxir but resistant to ranolazine or TMZ (Fig. 7A).

We finally examined whether the assay could be useful in analyzing FAO activity and regulation *in vivo*. After treatment with etomoxir, ranolazine or TMZ for 3 consecutive days, oxidation of administered ^3^H- palmitic acid was determined by measuring ^3^H_2_O present in plasma samples. Since ^3^H-palmitic acid, unlike freely circulated ^3^H_2_O, was not evenly distributed between tissue masses and blood circulation (data not shown), it was not possible to calculate the radioactivity of unoxidized ^3^H-palmitic acid by measuring the blood radioactivity only. Hence, we turned to ^3^H_2_O-based radioactivity present in the blood instead of the conversion ratio as an alternate index of *in vivo* FAO activity. As shown in Fig. 7B, the experiment confirmed that only etomoxir but not ranolazine or TMZ inhibited ^3^H_2_O production from oxidation of ^3^H-palmitic acid *in vivo*.

## Discussion

In this study, we described a detailed method to quantify FAO in mammalian cells and in animals. The assay was built on the principle that ^3^H_2_O and the FAO substrate ^3^H-palmitic acid can be separated on the basis of their differential diffusion rates. The versatility of the method was interrogated in established cell lines, primary cells and mice and validated with pharmacological and molecular approaches to depleting key FAO enzymes. Thus, the study provides a convenient and quantitative FAO assay and a clear picture of biological properties of potential FAO modulators in the mammalian system.

Different methods have been used to study cellular FAO activities. One strategy is to analyze metabolic intermediates of the FAO pathway with mass spectrometry^14,15^. The expression and activities of key FAO enzymes and regulators have been also employed as a reflection of FAO functionality^10,12,13^. However, the overall cellular FAO rate is not necessarily proportional to abundances of metabolic intermediates or expression/activity of individual FAO enzyme. Another approach is to determine oxygen consumption rates (OCR) in cells incubated with an assay medium containing palmitate as the sole source of oxidation substrate^16,17^. However, the OCR readouts are not direct measures of FAO.

Direct measurement of FAO by quantifying the conversion of ^3^H or ^14^C-labeled long-chain fatty acids to ^3^H_2_O or ^14^CO_2_ was originally developed for preliminary screening of inborn errors of FAO^31,40,63–66^. The ^14^CO_2_ release assay, however, has not been widely accepted as an accurate measurement given the low ^14^CO_2_ recovery rate, the wide normal range and large inter-assay variability commonly encountered^31^^,^^48^. In comparison, ^3^H_2_O release from the oxidation of tritiated palmitic acid offers a number of advantages. Among them are the higher specific activity detectable from smaller numbers of cells and the cost effectiveness^31,40^. In fatty acid substrates with tritium distributed equally between two adjacent carbon atoms, such as [9,10-^3^H(N)]-palmitic acid, greater than 75% of ^3^H label is eventually converted to ^3^H_2_O via the FAO pathway^31^. Conventionally, the assay involves a labeling buffer instead of complete medium to minimize lipid and protein contents for the convenience of the subequent separation of ^3^H-palmitic acid from ^3^H_2_O^20,31,32^. The physical separation is a labor intense procedure consisting of acid precipitation, neutralization, preparation of ion exchange resin columns, sample loading and water elution. These complicated steps are avoided in our diffusion method described here. Multiple samples can be readily analyzed simultaneously, making it especially useful for high throughput screening of potential FAO inhibitor and activators. The smaller number of error-prone manual steps also contributes to the accuracy and reproducibility of our assay. We have noted very consistent results from experiment to experiment if similar assay conditions were followed.

Although a number of structurally diverse compounds are considered to be pharmacological inhibitors of certain FAO enzymes^4,11^, no study has comprehensively examined their functional effects on the overall FAO activity with appropriate controls in intact cells. Physiologically, only the CPT system is considered to be the rate-limiting factor of the FAO pathway^67^. Partial inhibition of other FAO enzymes such as 3-KAT by ranolazine or TMZ may not be adequate to simulate FAO defects on the cellular level. More recent evidence has pointed to the other cellular targets for these antianginal drugs such as mitochondrial calcium channels as the mechanisms of their medical benefits instead of inhibiting FAO to improve myocardial glucose oxidation^25–29^. Consistent with this, our analyses did not reveal any FAO inhibitory effects of ranolazine and TMZ in established cell lines, primary cells and mice. Similarly, we did not detect an anti-FAO activity of perhexiline, a somewhat surprising observation as perhexiline was supposed to inhibit both CPT1 and to a lesser extent CPT2^46,47^. The lack of anti-FAO activity of perhexiline may be related to its poor specificity as a CPT1/CPT2 inhibitor^47^. Perhexiline has been reported to inhibit mTOR1, the delayed rectifier potassium channel and other cellular functions^68–70^. The broad spectrum of cellular targets may underlie the significant cytotoxicity associated with perhexiline and offset its FAO-interfering activity.

A major issue with the utility of etomoxir in the study of FAO is its inconsistent specificity and efficacy reported in the literature^2,17–20^. We therefore performed a detailed analysis of anti-FAO activities of multiple concentrations of etomoxir. Based on the results in many cell lines as well as in primary cells, we conclude that single-digital micromolar concentration of etomoxir is sufficient for maximal suppression of FAO in most cell types. The high doses of etomoxir used in many previous studies seem to be based on the assumption that only a limited fraction of etomoxir molecules are attached to CoA intracellularly to turn to an active CPT1 inhibitor^71^. By virtue of immunoprecipitation analysis, we provided compelling evidence that the etomoxir-CoA pool derived from a low micromolar concentration of etomoxir was abundant enough to saturate cellular CPT1 proteins, leading to potent inhibition of FAO activity. These results and recent discoveries of new, FAO-independent targets of etomoxir^18,21,72^ highlight the importance to select appropriate compounds and their functionally relevant concentrations for investigating biological functions of FAO.

Different isoforms of PPAR have been linked to upregulation of certain FAO enzymes or overall FAO activities in various cellular contexts^41,49,51,52^. Therefore, pharmacological antagonists of PPARs represent another class of FAO inhibitors that downregulate FAO via transcriptional repression of FAO pathway enzymes^41,52,53^. By assessing effects of antagonists of respective PPAR isoforms, we found that FAO in MCF-7 and T47D cells was most sensitive to the PPARα antagonist GW6471. This suggests that PPARα signaling is linked to the active FAO phenotype in breast cancer cells.

FAO modulators, especially selective inhibitors of FAO, are of great interest to the pharmaceutical industry^4^. In mammals, inhibition of FAO switches energy metabolism from fatty acids to glucose oxidation, a condition that typically alleviates insulin resistance and improves glucose metabolism^73^. Because of their hypoglycemic activity, certain FAO inhibitors, including etomoxir, were previously explored clinically for treatment of diabetes^74^. Although none of them passed clinical trials, the notion of developing specific FAO inhibitors to treat diabetes and other metabolic disorders remains attractive. More recently, emerging evidence suggests that the FAO pathway is abnormally activated and contributes to the malignant phenotype in various types of cancer^4,11^. Therefore, enzymes of the FAO pathway are now considered as novel anti-cancer targets. Our new FAO assay and updated knowledge on presently available FAO modulators will certainly facilitate the discovery of more potent and selective FAO inhibitors as potential anti-diabetes or anti-neoplastic agents.

## Materials and Methods

### Reagents

[9,10-^3^H(N)]- and [1-^14^C]-palmitic acids were obtained from Perkin Elmer (Boston, MA). Oxfenicine, perhexiline, ranolazine, TMZ, GW6471, GSK3787, GW9662, L-carnitine, AICAR, 2-DG, and sodium palmitate were from Sigma-Aldrich (St. Louis, MO). Essentially fatty acid-free BSA was obtained from Roche (Indianapolis, IN). Etomoxir and BAY-876 were purchased from Chemgood (Richmond, VA). FBS was from Atlanta Biologicals (Atlanta, GA). Glucose and cell culture reagents were obtained from Thermo Fisher Scientific (Waltham, MA).

### Cells

U-937, MCF-7, T47D, and MDA-MB-468 cell lines were obtained originally from the American Type Culture Collection (ATCC) (Manassas, VA). These cells were cultured in RPMI 1640 (2 g/l glucose, for U-937 and T47D) or DMEM (1 g/l glucose, for MCF-7 and MDA-MB-468) supplemented with 10% FBS, 100 U/mL penicillin, and 100 µg/mL streptomycin. Primary cardiomyocytes from adult mice were isolated by a collagenase-perfusion method as previously described^75^ and cultured in MEM supplemented with 10% FBS, 1 g/l glucose, penicillin/streptomycin, and 10 mM butanedione monoxime in laminin-coated plates. Primary hepatocytes from adult rats were obtained from Thermo Fisher Scientific (#RTCS20) and cultured in collagen-coated plates in Williams E medium containing 2 g/l glucose, penicillin/streptomycin, dexamethasone (0.1 μM), and thyroxine (1 μM). The cells reached 50-70% confluency at the time of FAO assay.

### FAO measurement

The cellular FAO activity was determined by quantifying the conversion of ^3^H palmitic acid to ^3^H_2_O over the labeling time. Cells were seeded in 12-well plates and when appropriate, treated with FAO inhibitors or stimulators. For FAO assays in complete medium with 10% FBS, [9,10-^3^H(N)]-palmitic acid [0.5 µCi (~9.3 pmol)] in 15 µl carrier solution (sodium palmitate 1 mM/BSA 0.17 mM/NaCl 150 mM)^76^ was added to each well (final concentration of added palmitate was ~15 µM, in addition to free fatty acids and other lipids present in FBS). For FAO assays in Krebs buffer or culture medium without FBS, the volume of carrier solution was increased to 50 µl, making a final concentration of palmitate at ~50 µM. After labeling, culture medium (0.5 ml out of 1 ml) was collected into a 15-ml Falcon polypropylene conical tube (#352096, BD Biosciences, San Jose, CA), and mixed with 100 µl of 1.2 N HCl to terminate all biological reactions. The 15-ml Falcon tube has a longer screw top to ensure tight closure than similar products of other brands. A 0.5-ml microcentrifuge tube containing 0.25 ml of sterile distilled water was uncapped and inserted into the 15-ml tube with forceps. Precautions were taken to prevent direct contact between the medium and water phases. The 15-ml tubes were tightly capped to allow diffusion between two liquid phases at room temperature for 3 days unless otherwise indicated.

To control for background from diffusion of ^3^H-palmitic acid, each experiment included a cell-free blank control with the same medium and water settings. When the diffusion of ^3^H_2_O reached equilibrium between the water and medium, 0.25 ml water or 0.25 ml medium (out of 0.6 ml) were mixed with 3 ml of scintillation cocktail and the radioactivities present in the 0.25 ml water (*a*) and 0.6 ml medium (*b*) were determined by scintillation counting. The ^3^H_2_O-based radioactivity in the whole sample would be $\frac{(250\,ul+600\,ul)}{250\,ul}$ × *a* = 3.4***a***, where the total radioactivity of the sample would be ***a*** + ***b***. The conversion of ^3^H-palmitic acid to ^3^H_2_O was calculated from the formula $\frac{3.4{\boldsymbol{a}}}{{\boldsymbol{a}}+{\boldsymbol{b}}}$. By subtracting the mean of the control group, the FAO rate for each sample would be ${(\frac{3.4{\boldsymbol{a}}}{{\boldsymbol{a}}+{\boldsymbol{b}}})}^{sample}-{(\frac{3.4{\boldsymbol{a}}}{{\boldsymbol{a}}+{\boldsymbol{b}}})}^{blankmean}$. The values were normalized to cell numbers and labeling times, and presented as % conversion/2 × 10^5^ cells/5 hours in most cases or % conversion/2 × 10^5^ cells/2 hours for the metabolically active hepatocytes and cardiomyocytes.

### Knockdown of FAO genes with lentivirus-mediated shRNA

The shRNA lentiviral vector pGreenPuro™ (System Biosciences, Mountain View, CA) was used to knock down the key FAO enzymes CPT1A and HADHA. The following were the shRNA target sequences: 5′-GGGAGTACGTCATGTCCATTG-3′ for CPT1A-sh^20^; 5′-TCTGTGAATCTCAGAAATTTG-3′ for HADHA-sh1; 5′-CCTGAGAAGGTGATTGGCATG-3′ for HADHA-sh2. As described previously, the shRNA sequences were cloned into pGreenPuro™^20^. The negative control was the pGreenPuro™ vector backbone that harbors a scrambled insert sequence (5′-CCTAAGGTTAAGTCGCCCTCG-3′). Recombinant lentivirus was produced by co-transfection into 293TN cells with lentiviral vectors and packaging plasmids using lipofectamine 2000 (Thermo Fisher Scientific). Supernatants were collected 48 hours after transfection and used to infect cells. Forty-eight hours post-infection, the transduced cells were selected with puromycin for additional 5 days before FAO assays.

### Immunoblotting and immunoprecipitation

Cells were lysed in RIPA buffer with Triton X-100 supplemented with protease/phosphatase inhibitor cocktail (Roche)^77^. Protein concentrations were measured with the Pierce BCA Protein Assay Kit (Thermo Fisher Scientific). Equal amounts of proteins were resolved by SDS-PAGE. The antibodies against CPT1A (ab128568) and CPT1B (ab134988) were obtained from Abcam (Cambridge, MA). The antibodies for β-actin (#4970) and glyceraldehyde 3-phosphate dehydrogenase (GAPDH) (#5174) were from Cell Signaling Technology (Danvers, MA). The antibody against HADHA (#PA5-27348) was obtained from Thermo Fisher Scientific. Immunocomplexes were visualized by an enhanced chemiluminescence detection kit (Thermo Fisher Scientific) using horseradish peroxidase-conjugated secondary antibodies (Cell Signaling Technology).

Cellular proteins (100 μg) from MCF-7 and T47D cells treated with or without etomoxir were immunoprecipitated with 3 μg anti-CPT1A antibody (ab128568) and 20 μl Dynabeads™ Protein G (Thermo Fisher Scientific). The immunoprecipitates and original cell lysates (input control) were separated by SDS-PAGE and blotted with the same CPT1A antibody as described above.

### Animals

All methods were carried out in accordance with relevant guidelines and regulations of Virginia Commonwealth University (VCU). All experimental protocols were approved by VCU Institutional Animal Care and Use Committee (IACUC). The *Cpt1a*^*fl/+*^ mice were obtained from Taconic Biosciences (Rensselaer, NY). The line was generated by introducing *loxP* sites flanking exon 10 via homologous recombination in C57BL/6 embryonic stem cells. The animals were intercrossed to obtain mice or embryos homozygous for *loxp Cpt1a (Cpt1a*^*fl/fl*^*)*. MEFs were prepared from embryos bearing *Cpt1a*^*fl/fl*^ or *Cpt1a*^*+/+*^ at embryonic day 14 as we described previously^78^.

The *Cpt1a*^*fl/+*^ mice were crossed with the *LysMcre* transgenic line (Jackson Laboratory)^42^ to generate myeloid lineage–specific deficiency in *Cpt1a (Cpt1a*^*fl/fl*^*LysMcre)* or the wild type control *(Cpt1a*^*+/+*^*LysMcre)*. Bone marrow-derived macrophages (BMDMs) from these mice were collected by flushing the marrow cavity of femurs. The cell suspensions were passed through a 22-gauge needle to disperse cell clumps. Cells were first cultured with DMEM supplemented with 10% FBS and 20% conditioned medium from L929 cells, a source of M-CSF to induce differentiation^79^. At day 6–8, BMDMs were subcultured and used for the FAO analysis.

To test whether the diffusion method could be extended to measure FAO activity *in vivo*, adult female C57BL/6 mice were treated for 3 days with etomoxir (i.p. 30 mg/kg/day), ranolazine (oral, 35 mg/kg/day) or TMZ (i.p. 40 mg/kg/day). On the last day, the mice were i.p. injected with [9,10-^3^H(N)]-palmitic acid (20 μCi in 200 μl carrier solution). The mice were euthanized 1 hour after the isotope injection and blood immediately collected into heparin-containing tubes (BD Vacutainer). Plasma samples were prepared by centrifugation at 2000g at 4 °C for 15 minutes. Cleared plasma samples (100 μl) were mixed with 400 µl PBS and 100 µl 1.2N HCl. The ^3^H_2_O-based radioactivity in the plasma samples was quantified with the diffusion setting as described earlier for culture supernatants.

### Statistical analysis

Statistical analysis was performed as we previously described^60^. All numerical data were presented as mean ± SD of triplicate assays. Statistical significances were determined using Student’s two-tail t-test, where *p* < 0.05 was considered statistically significant. The statistical significances were indicated with n.s. (not significant) if *p* > = *0.05*, * if *p* < 0.05, or ** if *p* < 0.01.

## Supplementary information

Supporting information.
