# Supplementary material for: Functional analysis of molecular and pharmacological modulators of mitochondrial fatty acid oxidation
Source: Sci Rep. 2020 Jan 29;10:1450. doi: 10.1038/s41598-020-58334-7 (PMC6989517; doi:10.1038/s41598-020-58334-7)
Supplement: Supplementary file 1 — Supporting information. [file 41598_2020_58334_MOESM1_ESM.pdf]

Functional analysis of molecular and pharmacological modulators of mitochondrial fatty acid oxidation

Yibao Ma[1], Wei Wang[1], Teja V. Devarakonda[2], Huiping Zhou[3], Xiang-Yang Wang[4], Fadi N. Salloum[2], Sarah Spiegel[1], Xianjun Fang[1,*]

From the [1]Departments of Biochemistry & Molecular Biology, [2]Internal Medicine/Cardiology Pauley Heart Center, [3]Microbiology & Immunology, and [4]Human & Molecular Genetics, Virginia Commonwealth University School of Medicine, Richmond, Virginia, 23298

[*]To whom correspondence should be addressed: Xianjun Fang, Department of Biochemistry & Molecular Biology, Sanger Hall 2-016, Virginia Commonwealth University School of Medicine, 1101 East Marshall Street, Richmond, VA 23298

E-mail: xianjun.fang@vcuhealth.org; Tel: 804-828-0787; Fax: 804-828-1473

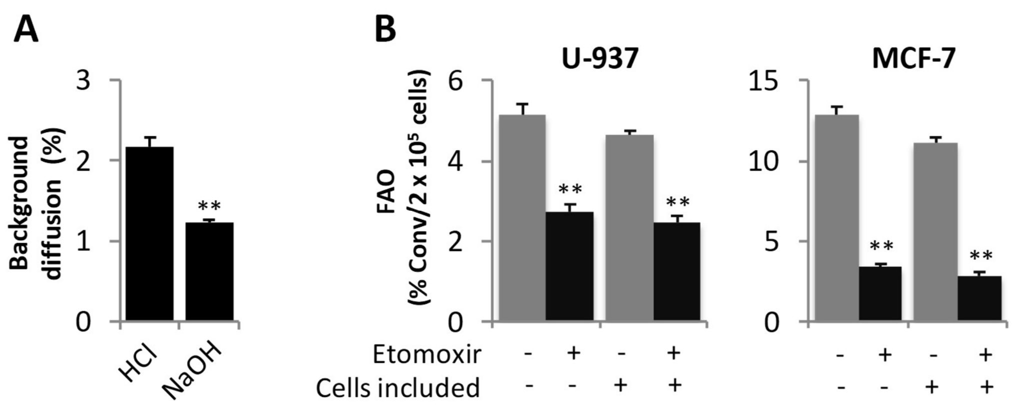

Supplementary Figure S1. Characterization of the FAO assay: (*A*) Comparison of the effects of acidic and base conditions on the background diffusion of ^{3}H palmitic acid. Complete culture medium in the absence of cells was incubated with ^{3}H-palmitic acid for 5 hours before NaOH (0.2N) or HCl (0.2N) was added and the diffusion device set up. The background diffusion rates were determined and calculated as detailed in Materials and Methods. **(*B*) FAO analyses using culture supernatants or combination of supernatants and cells.** FAO rates from analysis of culture supernatants with or without cells were measured and compared. U-937 and MCF-7 cells were treated with vehicle or etomoxir (5 μM) for 24 hours and labeled with ^{3}H-palmitic acid for the last 5 hours.

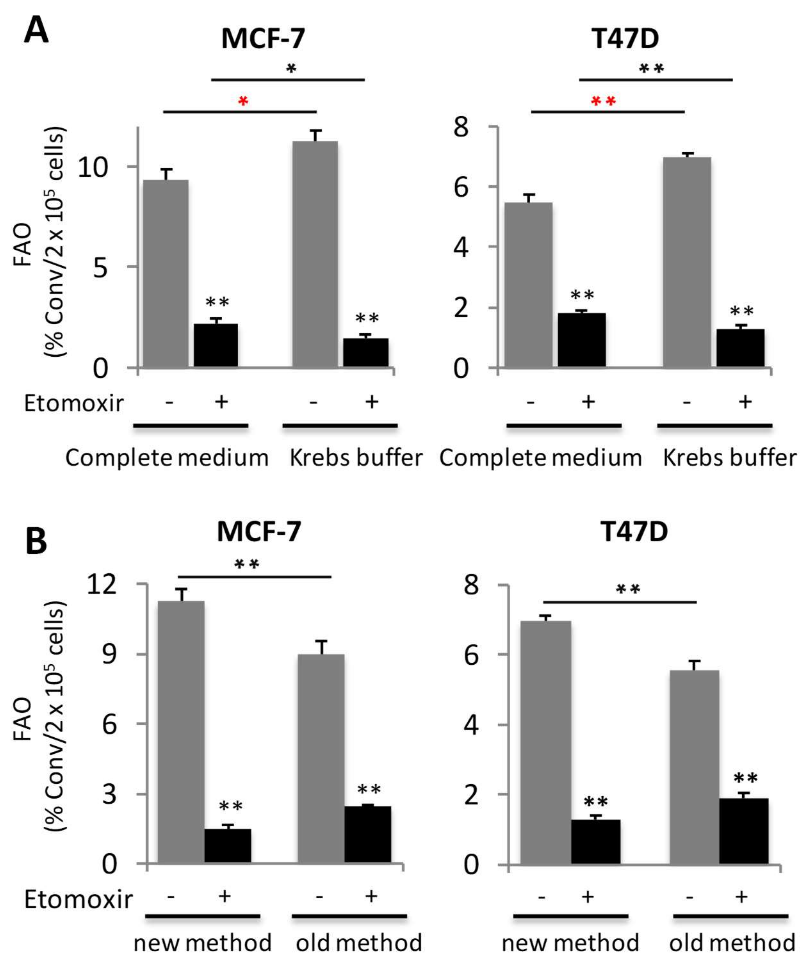

Supplementary Figure S2. Characterization of the FAO assay: (*A*) Comparison of FAO rates in cells labeled in complete medium or in Krebs buffer. MCF-7 and T47D cells were treated in complete medium with vehicle or etomoxir (5 µM) for 24 hours, followed by labeling with ^{3}H-palmitic acid in either complete medium or the Krebs buffer containing 1 g/l glucose for the last 5 hours before the diffusion analysis of FAO rates. (***B***) **Comparison of our new diffusion assay (new method) with the conventional physical separation method (old method)**. The cells were treated as described in (***A***) and then switched to the Krebs buffer for labeling with ^{3}H-palmitic acid for the last 5 hours. The conditioned buffer was subjected to analysis with both methods. The lipid precipitation, neutralization and column separation of $^{3}H_2O$ was performed as previously described (ref 31 & 32).

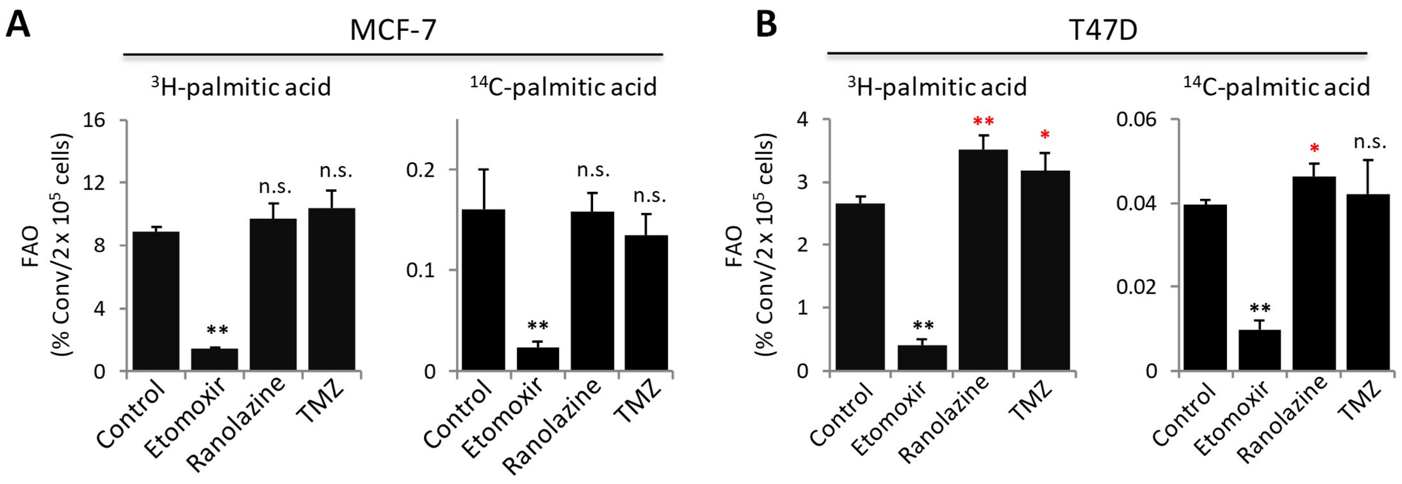

Supplementary Figure S3. Characterization of the FAO assay: Comparison of the ^3H_2O diffusion assay with the $^{14}CO_2$ capture method. MCF-7 (***A***) and T47D (***B***) cells treated with etomoxir (5 µM), ranolazine (25 µM), or TMZ (50 µM) for 24 hours were labelled in complete medium with [9,10-^{3}H]-palmitic acid (0.4 µCi) or [1-^{14}C]-palmitic acid (0.4 µCi) for the last 5 hours in complete medium. The $^{14}CO_2$ present in medium was captured for 2 hours with 1N NaOH solution (ref 66) and then radioactivities in the NaOH solution and medium phase were measured by liquid scintillation counting. The results from both methods were presented as FAO rates as defined in Materials and Methods, reflecting the conversion to ^3H_2O or $^{14}CO_2$ from the corresponding radioactive palmitic acid.

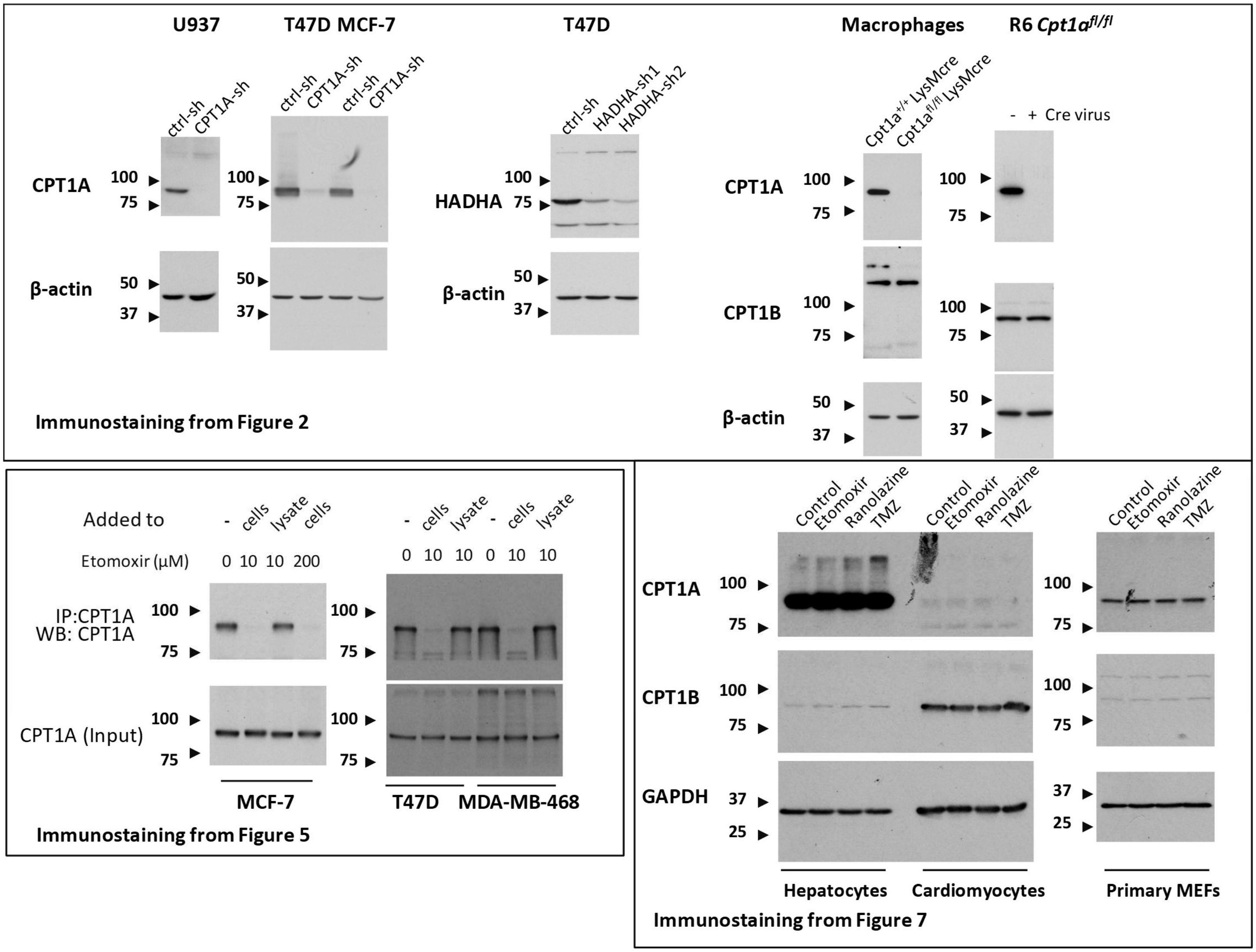

Supplementary Figure S4. Full-length blots from figures 2, 5 and 7 are presented.
